# Effects of Vemurafenib ± Cobimetinib on Intratumoral and Host Immunity in Patients With BRAFV600 Mutant Melanoma: Implications for Combination With Immunotherapy

**DOI:** 10.1002/cam4.71526

**Published:** 2026-01-09

**Authors:** Suthee Rapisuwon, Craig L. Slingluff, Jennifer A. Wargo, Ryan J. Sullivan, Benjamin Izar, Jia‐Ren Lin, Ileana S. Mauldin, Walter C. Olson, Christine A. Tran, Gabrielle H. Schwartzman, Geoffrey T. Gibney, Waddah Al‐Refaie, Michael B. Atkins

**Affiliations:** ^1^ Georgetown University‐Lombardi Comprehensive Cancer Center Washington DC USA; ^2^ University of Virginia Cancer Center Charlottesville Virginia USA; ^3^ University of Texas MD Anderson Cancer Center Houston Texas USA; ^4^ Massachusetts General Hospital Boston Massachusetts USA; ^5^ Columbia University Medical Center, Herbert Irving Comprehensive Cancer Center New York New York State USA; ^6^ Laboratory for Systems Biology Harvard Medical School Boston Massachusetts USA; ^7^ Creighton University School of Medicine and CHI Health Omaha Nebraska USA

**Keywords:** BRAF/MEK inhibitor therapy, melanoma, tumor immune microenvironment

## Abstract

**Background:**

Prior studies in patients with *BRAF*‐mutant melanoma have shown increased density of tumor infiltrating lymphocytes (TIL) after 2 weeks of BRAF (BRAFi) ± MEK inhibition (MEKi), but did not characterize the functional state or clonal diversity of TIL over time. We evaluated sequential tumor biopsies during therapy to test the hypotheses that BRAF/MEKi would increase TIL to day 29, with increases in IFNγ signatures and T‐cell homing receptor ligands and expansion of functional intratumoral tumor‐reactive CD8 T‐cells and TIL clonality in the tumor microenvironment.

**Methods:**

Subjects with biopsy‐accessible *BRAF*‐mutant advanced melanoma received vemurafenib+/−cobimetinib. Tumor biopsies were obtained at baseline and days 8, 15, and 29 on therapy. Tumors were analyzed by quantitative immunofluorescence (QIF), NanoString, and TCRseq.

**Results:**

Five patients were enrolled. All had an initial tumor response followed by subsequent progression. In four patients, both CD8^+^ and CD4^+^ TIL density increased by day 8 or 15 per QIF and continued to increase at day 29 in two. Gene expression data showed upregulation of genes/pathways associated with immunologic rejection of cancer, including Class I and II MHC expression, antigen processing/presentation, and critical T‐cell attracting chemokines. TCRvβ clonal expansion was observed in 3 patients, but most diminished after day 15.

**Conclusions:**

Data from this study provides provocative evidence that, while BRAF+/−MEK inhibitor therapy produces an increase in overall and clonal T cell infiltrates, there is limited evidence for generation of new or persistent tumor immunity. Thus, BRAFi/MEKi therapy may enable tumor‐reactive T cells to infiltrate tumors but tumor control does not appear to depend on priming new immune responses.

## Graphical abstract

We evaluated sequential tumor biopsies during therapy to test the hypotheses that BRAF/MEKi would increase TIL and lead to an expansion of functional intratumoral tumor‐reactive CD8 T‐cells and TIL clonality in the tumor microenvironment. Data from this study provides provocative evidence that, while BRAF+/−MEK inhibitor therapy produces an increase in overall and clonal T cell infiltrates, there is limited evidence for generation of new or persistent tumor immunity. Thus, BRAFi/MEKi therapy may enable tumor‐reactive T cells to infiltrate tumors but tumor control does not appear to depend on priming new immune responses.

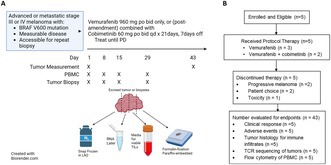

## 1 Background

In preclinical models and in patients with *BRAF‐mutant* melanoma, treatment with BRAF inhibitors (BRAFi) increased intratumoral CD4^+^ and CD8^+^ lymphocytes [1]. Also, in a murine syngeneic tumor model, inhibition of BRAF signaling enhanced production of IFNγ and TNFα [2] and increased MHC class I and II expression on tumor cells [3]. Further, in a murine model of adoptive T cell transfer, tumor‐reactive T cells homed more effectively to the tumor in the setting of BRAFi therapy, leading to increased tumor lysis [4]. In a subsequent clinical study, BRAFi ± MEKi increased intratumoral CD8^+^ T cell density, cytotoxicity‐associated proteins, and melanoma differentiation antigen expression, as well as decreased immunosuppressive cytokines within the first 2 weeks of treatment [5]. These studies suggested that the Mitogen‐activated Protein Kinase (MAPK) pathway contributes to tumor immune escape and that its inhibition may unleash antitumor immunity. However, these studies of the tumor immune microenvironment (TIME) were performed primarily at a single time‐point 1–2 weeks after initiating BRAFi ± MEKi [1, 5]. Contrastingly, at the onset of tumor progression, T cell density decreased and T cell exhaustion increased [5]. Thus, little is known about the time‐course of changes in the TIME between those time‐points.

Progressing melanomas create barriers to T cell infiltration that may explain the failure of immune checkpoint blockade (ICB) therapy with PD‐1/PD‐L1 antibodies (Abs) [6, 7, 8, 9]. The ability of BRAFi therapy to overcome those barriers, even if transiently, raised the possibility that co‐administering BRAF/MEKi with ICB would lead to synergistic anti‐tumor immunity. Clinical trials of such combinations have revealed increased objective response rates and increased progression‐free survival, but as yet no evidence of significant increase in OS compared to initiating treatment with ICB alone [10, 11, 12, 13, 14]. This modestly increased anti‐tumor efficacy appears not to represent significant immune synergy, but rather merely additive or even sub‐additive effects of two distinct treatment approaches.

Despite excitement generated around the potential relationship between MAPK pathway inhibition and enhanced antitumor immunity, critical details and the kinetics for this effect have not been fully characterized. For example, it is unclear to what extent the increased TIL density results from an expansion of specific subsets of tumor‐reactive T cells versus selective destruction of tumor cells and contraction of the tumor volume. It is also unclear whether the increased infiltrates peak at week 2 and dissipate before disease progression or whether disease progression precedes the decrease in infiltrates. To address these questions, we conducted a pilot study to evaluate serial tumor biopsies and blood samples through the first 4 weeks of vemurafenib ± cobimetinib therapy. Temporal changes in immune cell populations within the TIME were characterized and correlated with changes in peripheral blood and with clinical outcome. We hypothesized that BRAF/MEKi would (a) increase TIL to day 29, (b) increase IFNγ signatures, (c) increase T cell homing receptor (HR) ligands, and (d) expand functional intratumoral tumor‐reactive CD8 T cells, but also that (e) increased T cell infiltrates would be associated with increased expression of PD‐L1, IDO, and arginase by melanoma and stromal cells.

## 2 Methods

### 2.1 Study Design

**FIGURE 1:**
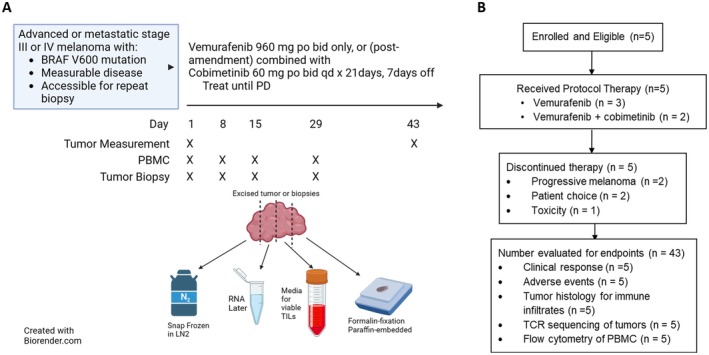
Graphical study protocol and CONSORT diagram.

The study was an open‐label, single‐arm phase II biomarker‐based trial in which patients with biopsy‐accessible advanced or metastatic *BRAF*‐mutant melanoma (Figure 1) received treatment with vemurafenib 960 mg orally twice daily alone. After a protocol amendment was approved, patients who were already on study could also receive after week (w) 4, cobimetinib 60 mg orally daily for 21 days (d) with a 7‐day (d) rest on a 28 days cycle, and newly enrolled patients would receive the combination of vemurafenib and cobimetinib from d1. Participants were treated until disease progression by Response Evaluation Criteria in Solid Tumor 1.1 (RECIST 1.1 [15]), unmanageable toxicities, or patient choice.

Key inclusion criteria were age at least 18 years, histologically confirmed metastatic melanoma harboring a *BRAF* ^*V600*^ mutation, as determined by an FDA‐approved assay, and measurable disease as defined by RECIST v1.1. Subjects were required to have ECOG performance status 0–1, adequate end‐organ function, and melanoma deposits of sufficient bulk and accessibility to enable four sequential tumor biopsies. Specifically, the minimum tumor size requirements for study eligibility were: (i) one lesion ≥ 5 cm^3^, (ii) two lesions ≥ 3 cm^3^ each, (iii) three lesions ≥ 2 cm^3^ each, or (iv) ≥ 3 skin lesions, such that the surface area was approximately 1 cm^2^ each (or in aggregate for several lesions) and the total volume of tumor was approximately 260–325 mm^3^ or greater for each biopsy timepoint. Key exclusion criteria included brain metastases treated less than 4 weeks prior to enrollment and prior systemic therapy for metastatic or advanced regional disease. Tumor measurements were performed by physical examination, computed tomography, and/or MRI every 8 weeks until w32, then every 16 weeks thereafter. Adverse events (AEs) were graded using Common Terminology Criteria for Adverse Events version 4.03.

Tumor biopsies were obtained at baseline (d1) and d8, d15 and d29. Each tumor biopsy was divided into five parts, with one part each placed in formalin, RNAlater, and O.C.T. tissue freezing medium, and two parts processed immediately for extraction of tumor infiltrating lymphocytes (TIL) which were then frozen at −80°C. Peripheral blood was collected concomitant with the biopsies, and peripheral blood mononuclear cells (PBMCs) were isolated by Ficoll and also frozen at minus 80°C for later analysis (Figure 1).

The primary endpoint was quantitation of CD8^+^ T cell infiltrates (per mm^2^ of tumor) over the first 4 weeks of treatment. Secondary endpoints included changes in the activation status and clonality of the TIL, expression of immune‐inhibitory proteins (including PD‐L1, IDO, and arginase) by tumors, and changes in immune‐responsive RNA signatures over the initial 4 weeks of treatment. Changes in immune cell numbers in the peripheral blood were also analyzed.

### 2.2 Characterization of the TIME and Circulating Lymphocytes

Formalin‐fixed, paraffin‐embedded (FFPE) blocks and fresh‐frozen PBMC and TIL were shipped from the study sites to Georgetown University for storage and later analysis.

### 2.3 Immunohistochemistry (IHC)

IHC analysis was performed on 5 μm sections of FFPE samples of the 4 tumor biopsies from Patient#1, with appropriate positive and negative controls and with primary antibodies for CD4, CD8, and FoxP3, followed by a secondary antibody for horseradish peroxidase and 3, 3′‐diaminobenzidine (DAB), respectively.

### 2.4 Multicolor Quantitative ImmunoFluorescence (mQIF)

mQIF was performed on unstained FFPE slides using the Perkin‐Elmer (now Akoya, Marlborough, MA) Opal system, according to the manufacturer's protocol with antigen retrieval buffers AR6, AR9 (Akoya), or Diva Decloaker (BioCare Medical, Pacheco, CA) as previously reported [16, 17, 18]. Opal Polymer HRP Secondary antibody was used (Akoya). Slides were mounted using prolong diamond antifade (Life Technologies, cat#P36961) and scanned at 10× magnification. Regions of interest (ROI) were identified in Phenochart software, and 20× magnification images were acquired. These images were spectrally unmixed using single stain positive controls and analyzed using the InForm software (Akoya) and a separate software algorithm developed at UVA for quantifying double‐stained cells. ROI representative of the tumor masses were identified, and staining was quantified. The assessed areas were summed for each specimen, and those areas served as the denominator for calculations of cell numbers per mm^2^. Also, the patterns of immune cell infiltrates were assessed to define Immunotypes, where Immunotype A represents sparse to absent infiltrates (less than 25 T lymphocytes/mm^2^), Immunotype B represents greater immune cell infiltrates but primarily confined to perivascular regions, and Immunotype C represents diffuse infiltration of immune cells within tumor cell nests, as described [19]. The cutoff of 25 T lymphocytes/mm^2^ instead of 50 CD45^+^ cells/mm^2^ in the original report was used because T cells represent ~50% of intratumoral CD45^+^ cells [19].

### 2.5 T Cell Receptor Clonality

Immunosequencing of the CDR3 regions of human TCRβ chains was performed using the ImmunoSEQ Assay (Adaptive Biotechnologies, Seattle, WA). Extracted genomic DNA was amplified in a bias‐controlled multiplex PCR, followed by high‐throughput sequencing. Sequences were collapsed and filtered in order to identify and quantitate the absolute abundance of each unique TCRβ CDR3 region for further analysis as previously described [20, 21].

### 2.6 RNA Expression Analysis in the TIME

RNA was isolated from the portion of the biopsy specimens frozen in the RNAlater (Qiagen) and analyzed with the nCounter PanCancer Immune Profiling Panel (NanoString Technologies Inc.) to evaluate the phenotype of the TIME. NanoString data were analyzed using the nSolver 4.0 Analysis Software and ROSALIND (San Diego, CA). Heat maps were generated using the *pheatmap*package v.1.0.12.

### 2.7 Flow Cytometry

Panels of fluorescently‐tagged murine monoclonal antibodies were prepared to examine immune cell sub‐populations in PBMC and in TIL. However, the number of viable TIL recovered from tumor biopsies was inadequate to perform phenotypic characterization by flow cytometry. Viably cryopreserved PBMC were thawed at 37°C and washed twice in pre‐warmed RPMI1640 containing 10% human serum (Type AB, Gemini). Cell viability was determined by trypan dye exclusion and a hemocytometer. Four antibody panels were used: panel 1: CD19, CD4, CD14, CD8, CD56, HLA‐DR, CD3; panel 2: CD45RO, CD27, CCR7, CD8, CD28, ICOS, CD3; panel 3: CD4, CD25, CD127, Ki67, FoxP3, CD3, CD45; panel 4: CD45RO, EOMES, PD‐1, t‐bet, CD3, CD8, TIM3. All panels also included Aqua as a live‐dead marker. Antibodies [and fluorescent tags] used in these panels are detailed in Table S1. Fluorescence‐activated cell sorting of PBMCs from each subject was performed on a BD FACSCanto II Flow Cytometer (Becton, Dickinson and Company BD Biosciences, San Jose CA) at baseline and on‐treatment. Using FlowJo software, version 10.8.1, PBMC were gated for CD3, CD4, and CD8 (CD3^+^CD4^−^) and assessed for expression of CD45RA and Ki67.

### 2.8 Statistical Methods

The target sample size estimation was 15 subjects. The primary statistical analysis was to assess if there were significant changes in CD8 T cell infiltration over time, with a paired *t*‐test, if approximate normality on the change (or its transformation) was achieved or a Sign test, if approximate normality could not be achieved. For the final analysis, a paired *t*‐test on square‐root transformed data was used. We had planned to use mixed effects models to study the change in CD8 T cell counts per mm^2^ of tissue, changes in expression of immune‐inhibitory proteins (PD‐L1, arginase), and tumor‐associated chemokines at pre‐treatment and at weeks 1, 2, and 4 after the initiation of vemurafenib ± cobimetinib therapy. Patients were to be treated as random effects to account for individual variability. Potential covariates included age, gender, and ECOG PS. However, due to premature study termination because of low accrual, the statistical methods of the biomarker data were altered to be primarily descriptive and exploratory.

### 2.9 Statistical Analyses of TCR‐β Sequencing Results

Clonality was defined as 1‐ Peilou's eveness [22] and was calculated on productive rearrangements by: $1+\frac{\sum_{i}^{N}p_{i}\log_{2}\left(p_{i}\right)}{\log_{2}\left(N\right)}$ where *p*_*i*_ is the proportional abundance of rearrangement i and N is the total number of rearrangements. Clonality values range from 0 to 1 and describe the shape of the frequency distribution: clonality values approaching 0 indicate a very even distribution of frequencies, whereas values approaching 1 indicate an increasingly asymmetric distribution in which a few clones are present at high frequencies. Statistical analysis was performed in R version 3.2.

For TCR‐β sequencing from FFPE tissues, the fraction of T cells in FFPE tissue samples was calculated by normalizing TCR‐β template counts to the total amount of DNA usable for TCR sequencing, where the amount of usable DNA was determined by PCR amplification and sequencing of several housekeeping genes that are expected to be present in all nucleated cells.

### 2.10 Analysis of TCR Sequencing Data

Multiple tools through Adaptive Biotechnologies' ImmunoSEQ analyzer were used for analysis. The Differential Abundance tool allows for identification of TCR sequence upregulation between two different time points. This tool was used to compare d1 pre‐treatment tumor samples with d8, d15, and d29 post‐treatment tumor samples to identify unique TCR sequences that were significantly increased (*p* < 0.05) post‐treatment. The Track Rearrangements tool was used to generate the TCR nucleotide sequences that were among the top 25 productive frequencies. The Venn Diagram tool was used to generate the number of overlapping nucleotide sequences across samples, which can be used for up to four samples.

## 3 Results

### 3.1 Demographics and Efficacy

The protocol was activated in 2013 with vemurafenib (BRAFi) monotherapy. It was later amended to administer the vemurafenib+cobimetinib combination therapy after three randomized phase III trials [23, 24] established greater efficacy for the BRAFi/MEKi combinations relative to BRAFi monotherapy. This necessitated IND filing with the FDA for cobimetinib (as cobimetinib was not yet FDA approved). The protocol was closed to accrual after five patients were enrolled. Three were treated with vemurafenib (BRAFi) alone and two with vemurafenib+cobimetinib (BRAFi+MEKi). All had an objective tumor response (1 complete response (CR), 4 partial responses (PR)). Reasons for discontinuing study treatment were patient choice(2), progressive disease(2), and toxicity(1). The average duration of treatment was 25.2 weeks (8–56 weeks). Demographic and clinical data are summarized in Table 1.

**TABLE 1:** Demographic and clinical information.

Subject #	Age group	Gender	Treatment	RECIST best response	Duration of response (weeks)	Reason off study
1	55–59	Female	V	PR	8+	Patient choice
2	30–35	Male	V	PR	12	PD
3	70–75	Male	V + C	CR	56+	Toxicity and non‐compliance
4	60–65	Male	V	PR	n/a	Patient choice
5	55–59	Male	V + C	PR	42	PD‐ brain mets

*Note:* All subjects who withdrew from the study were deemed to be unrelated to any treatment‐related adverse events.

Abbreviations: C, cobimetinib; CR, complete response; PD, progressive disease; PR, partial response; RECIST, Response Evaluation Criteria In Solid Tumors; V, vemurafenib.

### 3.2 Adverse Events

Grade 3 treatment‐related AEs are summarized in Table S2. There were no grade 4 AEs or deaths observed in this study. The one toxicity‐related treatment discontinuation was due to grade 3 elevated liver function tests and subretinal edema.

### 3.3 Evaluation of Immune Infiltrates in Metastases

**FIGURE 2:**
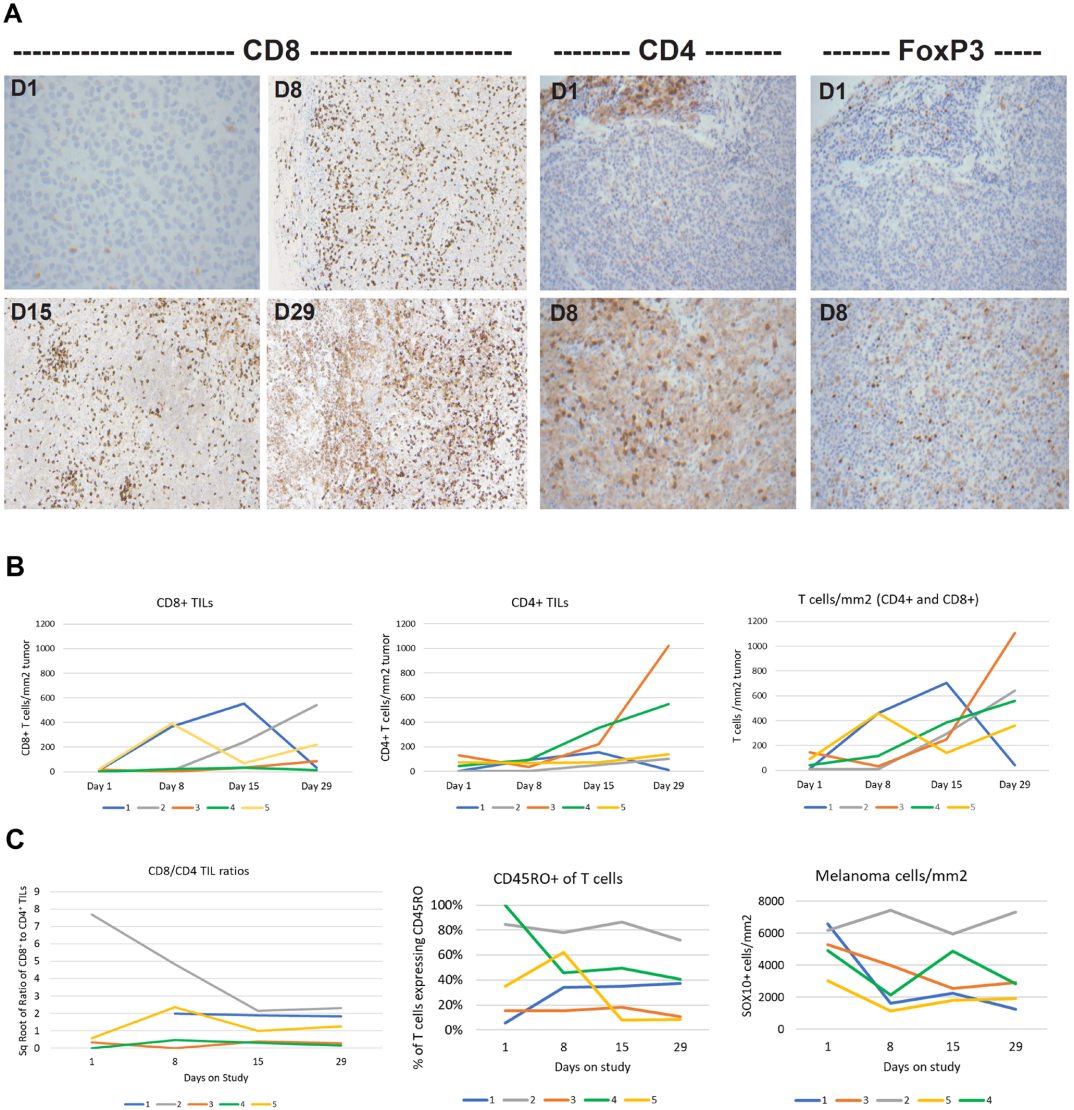
Density of TILs subsets at baseline and during the first 4 weeks of BRAF/MEK targeted therapy. (A) CD8, CD4, and FoxP3 cells detected by IHC in melanoma of patient 1; (B) Density of CD8^+^ TILs, CD4^+^ TILs, and total TILs (CD8^+^ and CD4^+^) by QIF: changes in total TILs were significant at day 15 (*p* = 0.041) and at day 29 (*p* = 0.016) by 2‐tailed paired *T*‐test of square root‐transformed data; and (C) square root of ratios of CD8^+^ to CD4^+^ TIL, percent of tumor infiltrating T cells expressing CD45RO, and changes in numbers of SOX10^+^ melanoma cells per mm^2^. The calculated % of CD45RO+ CD8+ cells was 108%, but was corrected to 100% for the graph.

For all 5 patients, the tumors submitted to sequential biopsies were all subcutaneous metastases with or without deep dermis involvement. Biopsy specimens from Patient#1 were evaluated by IHC staining for CD4, CD8, and FoxP3 as a proof‐of‐concept early after study initiation. This analysis identified increases in CD8^+^ lymphocytes from baseline peaking after 1 week and forming intratumoral lymphocytic aggregates suggestive of early tertiary lymphoid structures after 2 weeks (Figure 2A). The d29 biopsy specimen showed increased necrosis and pigment‐laden melanophages, but fewer CD8^+^ lymphocytes. Concomitant increases in CD4^+^ and FoxP3^+^ cells on treatment were observed (Figure 2A).

### 3.4 Multiplex Quantitative Immunofluorescence Histology

For the primary objective of this study, we determined the kinetics of changes in cell infiltrates during BRAFi+/−MEKi therapy. All 20 biopsy specimens from the five patients were subjected to mQIF. There was insufficient tumor remaining from d1 of Patient#1; so values for CD8, CD4, and FoxP3 infiltrates from the single‐color IHC were used as baseline values for this patient.

There was minimal or undetectable intratumoral CD8^+^ T lymphocytic infiltration at d1 from all patients, with 0–22 CD8^+^ cells per mm^2^, followed by marked increases in three of five patients (1, 2, and 5) by d15 (Figure 2B, left panel). The density of CD8^+^ cells subsequently decreased in two patients by d29, continued to increase in two others and did not change meaningfully for one patient (#4). CD4^+^ T cells at baseline ranged from 0 to 128/mm^2^ (median 70, mean 48) and increased to peak values ranging from 99 to 1021/mm^2^ (median 153, mean 392), where these values peaked at d15 for Patient #1 and d29 for two others (Figure 2B, middle panel). These changes in CD8 and CD4 T cell density were not significant due to small numbers and interpatient variability. However, the total percentage of T cell infiltrates (sum of CD4^+^ and CD8^+^ T cells) increased with BRAFi±MEKi over the 4 weeks, with statistically significant increases by d15 (two‐sided *T* test of square root‐transformed data, *p* = 0.045; mean = 169/mm^2^) and d29 (*p* = 0.038, mean 364/mm^2^) (Figure 2B, right panel).

To assess if there was selective expansion of CD8 T cells, CD8/CD4 ratios of TIL in the serial biopsies were compared. These ratios decreased from baseline in one patient (#2), due to very low CD4 counts at baseline that increased with treatment; however, they remained relatively consistent over time for the others (Figure 2C, left panel). There were some early changes in the percentage of antigen‐experienced (CD45RO^+^) T cells in the tumors, but no consistent pattern (Figure 2C, middle panel). Therapy was associated with early and dramatic decreases of melanoma cell density in 4 of 5 patients (SOX10; Figure 2C, right panel).

### 3.5 Immune‐Related mRNA Transcripts Analysis

**FIGURE 3:**
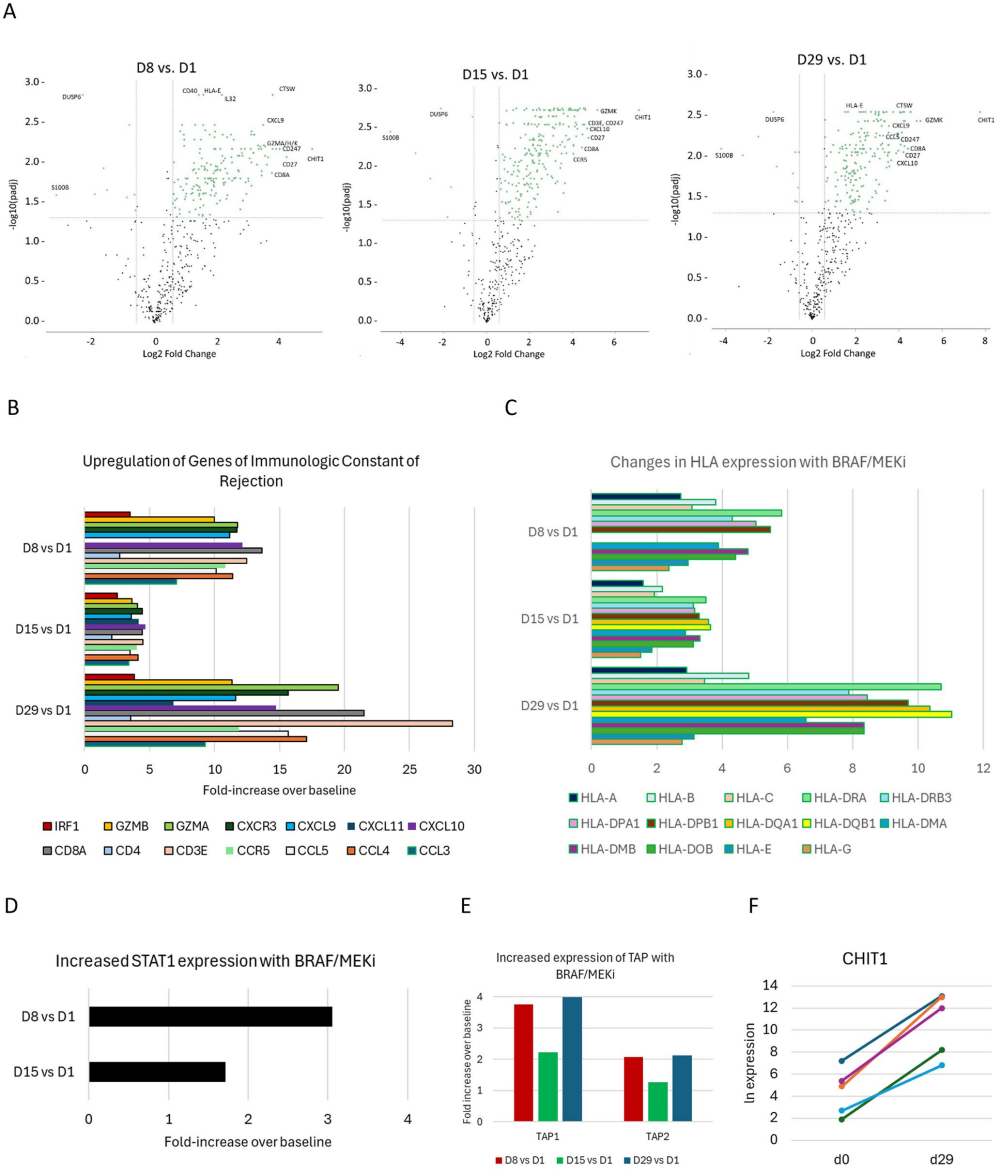
Changes in gene expression in the BRAFi/MEKi treated melanoma tumors over time, by Nanostring: (A) Volcano plots showing log_2_ fold‐change at D8, D15, or D29 versus D1, with adjusted *p* values plotted on a negative log_10_ scale. These are controlled for patient as a co‐variate. Some of the most significantly altered genes are marked with their gene names as examples. (B) fold‐increases over baseline at each time point for 14 genes in a Th1 pathway associated with immune rejection of cancer; (C) increases in HLA genes; (D) significant fold‐increases in STAT1 expression at D8 and D15; (E) significant increases in TAP gene expression; (F) expression of CHIT1 at baseline and D29.

Among the 5 patients, there was a variation in baseline expression of immune genes. The data were analyzed with the patient as a covariate so that changes over time could be assessed across the study population. As shown in volcano plots (Figure 3A), 252 genes were upregulated on at least one of the 3 timepoints (d8, 15, and 29) versus baseline, with 160 (63%) being significantly upregulated at all 3 timepoints. Details of these genes are provided in Table S3. The 20 genes most upregulated (based on median fold‐increase, among the 3 time points, and with adjusted *p* values < 0.05) were, in order, starting with the most upregulated, CHIT1, CD27, CD247, GZMK, CTSW, CD8A, CD3E, CXCL10, GZMA, CXCR3, CCL4, GZMH, CXCL9, CCR5, CD3D, LY9, CLEC7A, CD48, CCL5, GZMB. The full set includes gene markers for T cells (CD3, CD4, CD8), chemokines and chemokine receptors crucial for homing of activated T cells, and key genes for cytotoxic T cell function. Among those significantly increased with BRAF+/−MEKi were 14 of 17 genes of a Th1 signature associated with immunologic rejection of cancer [25] (Figure 3B), with GNLY, IFNG, and GBP1 the only genes not represented. Though IFNG (gene for IFNγ) was not significantly increased, STAT1 was significantly upregulated (Figure 3D and Table S3), and the IFNγ‐inducible chemokines CXCL9, CXCL10, and CXCL11 were all significantly upregulated (Figure 3B and Table S3).

There also was significant upregulation of Class I and Class II MHC genes (Figure 3C) and critical genes of antigen processing, including TAP1 and TAP2 (Figure 3E). The most consistently and dramatically upregulated was CHIT1 (chitinase 1), with greater fold‐increases by d29 than seen at d8 and d15. The fold‐increases for CHIT1 from baseline to d29 are shown for all 5 patients (Figure 3F).

Fourteen genes were significantly downregulated versus baseline with 5 (S100B, DUSP6, EGR1, GPI, and PVR) significantly downregulated at all 3 time points (Table S3). S100B downregulation is consistent with the observed destruction of melanoma cells, which are usually S100^+^. DUSP6 is a protein phosphatase that inactivates ERK [26]. EGR1 is a transcription factor that can have oncogenic roles in melanoma and other cancers and can support angiogenesis, but it can also enhance tumor cell apoptosis [27]. GPI can support metastasis, and PVR (CD155) is a ligand for TIGIT [28].

Accordingly, 21 immune‐related BioPlanet gene pathways were upregulated significantly (adjusted *p* < 0.05), the 7 most significant of which were adaptive immune system, antigen processing and presentation, viral myocarditis, cell adhesion molecules, immune system, antigen processing: cross‐presentation, and allograft rejection—all of which were significantly upregulated at d8, d15, and d29 (Table S4).

The Nanostring data also enabled identification of gene sets as markers for varied immune cell populations, using the Cell Atlas gene sets. Those that were significantly upregulated (FDR‐adjusted *p* values < 0.05) included CD8^+^T cells, CD14^+^ monocytes, whole blood, CD33^+^ myeloid cells, and BDCA4^+^ dendritic cells at all 3 time points (vs. d1), and increases in CD4^+^ T cells and CD56^+^ NK cells on d8 versus d1 only (Table S5).

Relevant to our hypothesis that there would be increased expression of checkpoint molecules, there were significant increases in CD274 (PD‐L1), PDCD1LG2 (PD‐L2), TIGIT, HAVCR2 (TIM‐3), but not PDCD1 (PD‐1), IDO‐1, or Arginase I (Table S3).

### 3.6 Expansion of T‐Cell Receptor Clonotypes in TIL

**FIGURE 4:**
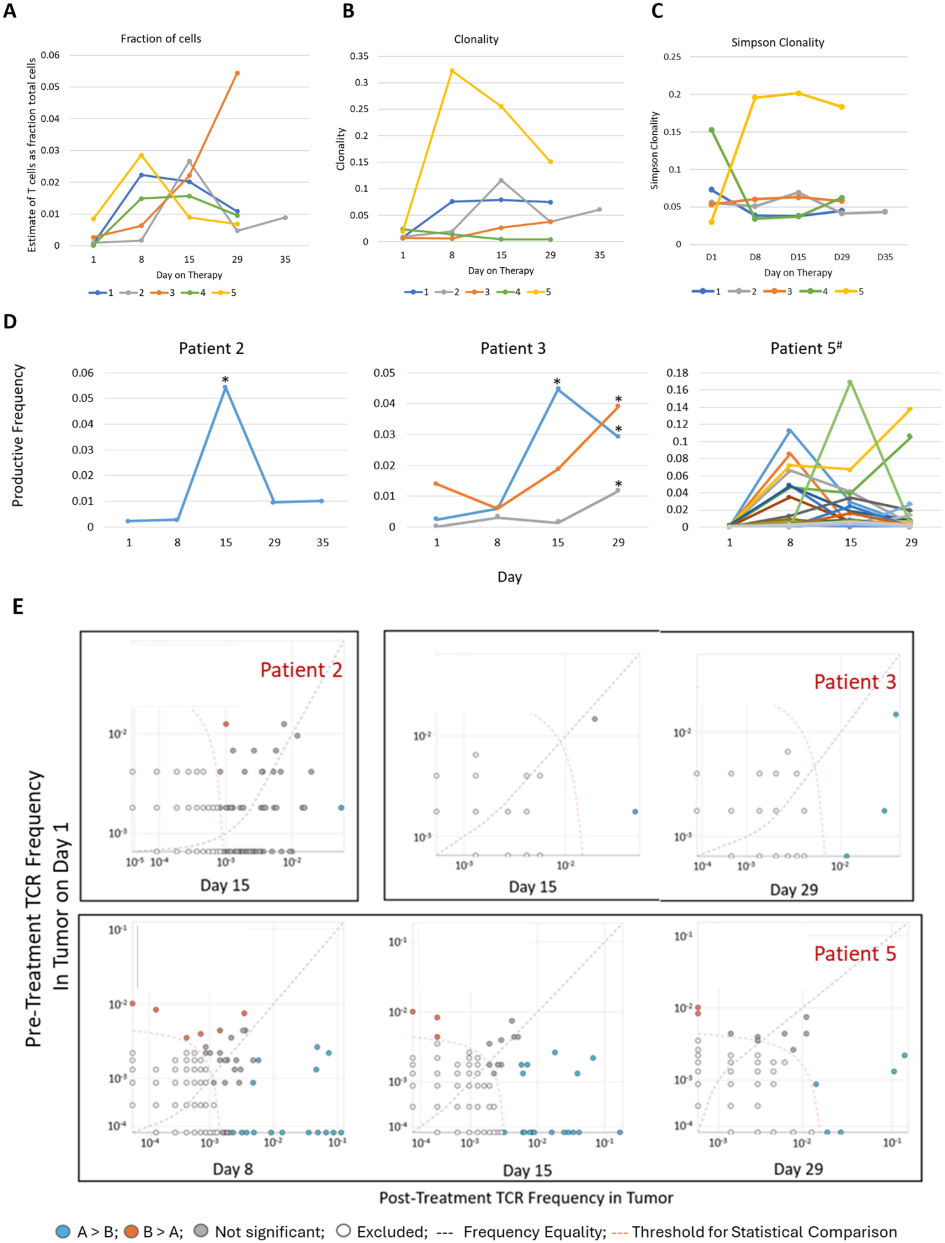
Expansion of TCR Clonotypes Post‐Treatment versus Pre‐Treatment. (A) changes in the estimated proportion of total cells containing productive T cell receptor rearrangements are plotted for all 5 participants, from days 1–29. (B) Productive TCR clonality is plotted for all 5 participants. (C) Simpson productive TCR clonality for all 5 participants. For three patients with significant expansion of one or more TCR clonotypes data are shown in (D): for each patient, each significantly increased TCR clonotype is represented by a unique color line. TCR clonotypes that were significantly expanded (*p < 0.05) at any point post‐treatment compared to pre‐treatment. ^#^For Patient 5, the large number of significantly increased TCR clonotypes are difficult to discriminate visually—details are in Table S6; (E) The frequency of each clonotype at D8, D15, or D29 is plotted versus the frequency at D1. Frequency equality pre‐treatment versus post‐treatment is represented by the gray dotted oblique line. The threshold for statistical comparison is represented by the red dotted curved line. Sequences represented by light gray were below this threshold and subsequently excluded from analysis. Sequences represented by orange appear in higher frequency D1 than post‐treatment. Those in blue are in significantly higher frequency post‐treatment (*p* < 0.05) versus D1. Sequences on the *x*‐axis are present post‐treatment, but absent pre‐treatment. Sequences represented by dark gray circles are not statistically significant (*p* > 0.05).

Serial assessment of TIL TCRvβ over time showed increases in T‐cells, as a fraction of total cells, in all samples starting from d8 of treatment (Figure 4A). There were increases in clonality for 4 of 5 patients (except #4), peaking at different time points among the patients (Figure 4B). Simpson clonality increased dramatically after d1 for patient #5, but declined from d1 to d8 for 3 patients, prior to increasing after d8 (Figure 4C).

TCR nucleotide sequences of the top 25 most productive clonotypes in tumors were graphed across all time points for each patient (Figure S1). Significant expansion of specific Vβ T cell clones was observed in 3 patients (Figure 4D,E). In Patient#2, clonal expansion of a TCR Vβ28‐01*01 clonotype was observed (0.4% at baseline to 5.4% on d15). In Patient 3, there was significant expansion of 3 Vβ clonotypes, the most prominent of which was TCR Vβ09‐01 (0.23% at baseline to 4.4% on d15). TCR Vβ composition in Patient#4 appeared oligoclonal at baseline, but the template abundance was low, which affected the composition of overall cellular infiltrate. Patient#5 TILs exhibited expansions of 27 distinct TCR Vβ clones, many of which were undetectable at d1 (Figure 4E); some of these peaked transiently at d8, others at d15, and two still were increasing to d29 where each represented more than 10% of the TIL (Figure 4D,E). Details of the CDR3 variable regions are provided in Table S6. Clonotype frequency plots for those without significant clonal expansion are provided in Figure S2. For Patients 2, 3, and 5, the significantly increased clonotypes represented 5.4%, 6.4%, and 51.4% of total TIL on d15, respectively. Those proportions were 1.0%, 8.0%, and 34.5%, respectively, on d29.

Interestingly, many TCR Vβ clonotypes were identified in all post‐treatment tumor biopsies but were undetectable in tumors pre‐treatment: the numbers of these clonotypes were 579, 81, 20, 12, and 39 for patients 1–5, respectively. These are represented in Figure S3.

### 3.7 Flow Cytometry Findings in PBMC

PBMC flow cytometry data were evaluable for 4 of 5 patients. While there were absolute increases in T cells, the results revealed no consistent changes in the proportions of T cell subpopulations (CD4^+^, CD8^+^, CD19^+^, CD28^+^, CD56^+^, Tbet^+^, EOMES^+^, CD45RO^+^, PD1^+^) over time. We also did not observe any distinct pattern of change in CD4^+^, CD8^+^, CD19^+^, CD19^+^HLADR^+^, or CD56^+^ lymphocyte numbers over time. Further, the proliferation index as measured by Ki67 also did not significantly change over time for circulating CD4 or CD8 T cells (Linear Regression: −0.00702 ± 0.00725; *p* = 0.435) (Figure S4).

## 4 Discussion

Combination BRAF/MEK inhibitor therapy has improved the progression‐free and overall survival of patients with BRAF^V600^ mutant metastatic melanoma relative to either dacarbazine or single agent BRAFi therapy [23, 24]. Prior studies have suggested that MAPK pathway inhibition may enhance antitumor immunity through a variety of mechanisms, including increased melanoma‐specific antigen presentation and reduction of immunosuppressive cytokines and regulatory T cells [5]. In this study, we examined the immunophenotypic and transcriptional characteristics of the tumor lymphocytic infiltration serially over the first 4 weeks of treatment. We observed increased TIL infiltration within the first 29 days of therapy, as well as increases in both CD4^+^ and CD8^+^ subpopulations within 8–15 days of treatment initiation, but the kinetics of change in CD8^+^ TILs varied among individuals, with some peaking d8 or 15, but others persisting through d29. The CD8/CD4 ratio did not change and a commensurate increase in FoxP3^+^ cells was also noted. Gene expression data show upregulation of genes and pathways associated with immunologic rejection of cancer [25], including increases in Class I and II MHC expression and antigen processing/presentation. Together, analysis of cellular infiltrates, gene expression, and TCR sequencing provides a mixed picture of the immunologic events in the TIME induced by BRAF+/−MEKi therapy. For some patients, there was clonal expansion of T cells through d29, but for most, that was either absent or transient. The data support increased MHC and TAP expression in the TME, strong upregulation of genes of Th1 function, and increases of critical T‐cell attracting chemokines. However, clonal expansion of T cells appears to be the exception rather than the rule.

Our data support prior studies showing increased TIL at 1–2 weeks after BRAF/MEKi therapy for advanced melanoma [1, 4, 29] and extend those findings by showing that this increase in TIL persists in most patients through the first month, including both CD8 and CD4 T cells. Prior work has shown that BRAF inhibition increases expression of melanoma‐associated antigens by melanoma cells [30], and our gene expression data also show increased expression of Class I MHC and Class II MHC in the TIME, which could be due to enhanced expression of MHC by melanoma cells, or the increase of T cells expressing both MHC Class I and II alleles. Also, we find increased expression of TAP1 and TAP2, which are critical to antigen processing and presentation. Accordingly, the antigen processing and presentation pathway is the 2nd most upregulated for all 3 on‐treatment timepoints. Increased expression of melanoma antigens by the melanoma cells is difficult to measure in these studies, as the therapy induced objective clinical responses in all patients, along with reduced numbers of viable melanoma cells during treatment.

The possible mechanisms for increasing TIL could include increased homing to the TIME or clonal expansion of TIL already in the TIME. Alternatively, the immune cell enrichment could be related to selective death of tumor cells over time and an artifactual increase in TIL density due to contraction of the tumor volume. Our data provide evidence in support of each of these mechanisms. For example, there is marked upregulation of critical T‐cell attracting chemokines CXCL9‐11 and CCL5 in these tumors, a finding evident at d8 and persisting through d29. Because CXCL9‐11 are induced by IFNγ, and because STAT1 expression is enhanced, it is likely that IFNγ expression is enhanced, despite its lack of definitive increase by Nanostring. IFNγ is induced early after antigen exposure, so it may have increased before d8, and then subsided as the downstream effects are realized. Regardless, the findings support increased homing of T cells into the melanoma TIME. For two of the patients, there is no evidence of clonal expansion of TIL; so, in those patients, T cells must be entering the TIME and not encountering cognate antigen. This raises the possibility that combining adoptive T cell therapy or cancer vaccines with BRAF/MEKi may be valuable for such patients to support clonal expansion in the TIME of melanoma‐reactive T cells.

Alternatively, 3 patients showed evidence of clonal T cell expansion, ranging from transient expansion of one TCRVβ T cell clonotype (Patient#2) to expansion of 3 TCRVβ T cell clones, which persisted through day 29 and represented 8% of TIL (Patient#3), as well as expansion of dozens of TCRVβ T cell clonotypes in Patient#5, where most expanded transiently, but a few persisted through d29. In Patient#5, the TIL expanding clonally in the TIME represented 50% of the TIL on d15 and over 33% on d29. While the specificity of these TIL is unknown, those that continue to undergo clonal expansion through d29 may well be specific for antigens expressed by the melanoma cells. Reasons for the transience of clonal expansions are unknown, but a possible explanation is that diminished tumor antigen presentation associated with tumor cell loss might have hampered further activation of clonal T cells. Alternatively, MEK inhibition might have prevented further T cell expansion. Nonetheless, these data suggest that some patients with pre‐existing T cell responses to tumor antigens in their TIL may benefit from a brief course of BRAF+/−MEKi therapy prior to initiation of ICB as was seen in the “Sandwich Therapy Arm” of the Secombit Trial [31].

Finally, some of the perceived T cell expansion and increased expression of immune genes may be due to the observed profound loss of tumor cells and resultant tumor mass shrinkage. Nanostring data showed significant downregulation of tumor‐associated genes relative to baseline including 5 genes at all 3 post‐baseline timepoints. Further, we observed expansion of both CD8 and CD4 cells (as well as FoxP3‐expressing cells in Patient#1) and no consistent expansion in the percentage of antigen‐experienced CD45RO^+^ cells, suggesting that some of the observed increased T cell density was non‐specific and could be artifactual.

Interestingly, the gene most dramatically and consistently upregulated during BRAF+/−MEKi therapy was CHIT1, a gene that is expressed in activated macrophages and associated with chronic inflammatory conditions [32, 33, 34]. Myeloid cells can have a range of roles in antitumor immunity, ranging from supporting antigen presentation to immune suppression. CHIT1 expression outpaces that of all other immune‐related genes from d8 to d29, leaving open the possibility that it may be an early harbinger of changes that occur later during BRAF/MEKi therapy. Assuming this finding is not a consequence of selective persistence of CHIT1‐expressing cells in the setting of BRAF+/−MEK inhibition, further studies of the cells expressing this gene may yield new targets to enhance immune function during mBRAF/MEKi therapy.

Taken together, these data suggest that T cell influx is not consistently due to generation of new antitumor immunity and that BRAF/MEKi therapy may, at best, augment an existing immune response rather than priming a new one. Further, despite the clear evidence of clonal CD8^+^ T cell expansion within the TIME extending out to d29 in some of our patients, the known lack of durable tumor responses to BRAF/MEKi, especially once treatment is stopped, suggests that this clonal expansion does not persist and is insufficient to eliminate the last tumor cell.

This study has several limitations. These include the small sample size, the slow accrual, and the fact that only 2 of the 5 enrolled patients received the current standard‐of‐care combination therapy with BRAF and MEK inhibition, while 3 received BRAF inhibition alone. Despite these limitations, all had objective clinical responses and four sequential biopsies of skin/subcutaneous metastases were obtained and evaluated for all five participants. Another limitation is that biopsies were only obtained through the first 29 days on therapy. This provides sequential information that extends prior observations from a single time point, but future studies may benefit from additional scheduled biopsies at later time points, especially at the time of tumor progression at the same site.

In summary, we identified the changes in TIL over time during BRAF+/−MEKi therapy and have interrogated temporal changes in immune cell populations within the TIME and in peripheral blood. Our analysis of these serial biopsy specimens early in the course of MAPK inhibitor therapy suggests mechanisms by which these drugs might enhance T cell infiltration and, in some patients, expand antitumor immunity and potentially point the way to enhancing these immune effects. These data also suggest that while combinations of BRAF/MEK inhibitors with anti‐PD1/PD‐L1 may increase the likelihood of antitumor effects, it is unclear that they will enhance the curative effects of anti‐PD1/PD‐L1 monotherapy. However, it should be noted that our sample size was quite limited and therefore these observations would benefit from confirmation in a larger patient subset. We also noted that, in some patients, there were clonal expansion and dramatic shifts in CD8/CD4 ratios, albeit transient. These findings suggest the need for other interventions to overcome tumor‐associated barriers to immune function (e.g., overcoming intratumoral T cell exhaustion). Lastly, the non‐uniformity of T cell function kinetics among the 5 patients, although possibly related to sampling bias, also suggests heterogeneity within the biology of MAPKi‐T cell interaction, which, if true, could necessitate personalized approaches towards treatment combinations.

In general, these data portend a lack of synergistic antitumor immune activation with concomitant BRAF/MEKi and ICB therapy and may explain the disappointing efficacy results in the various randomized trials comparing “triple therapy” to BRAF/MEKi therapy alone. Alternatively, the DREAMseq and SECOMBIT trials comparing the use of combination ICB (nivolumab plus ipilimumab) and BRAF/MEK inhibitors in sequence both showed superior OS outcomes with the initial use of immunotherapy compared to the converse sequence suggesting that this approach (rather than triple therapy) should be preferred for most patients with metastatic *BRAF*‐mutant melanoma [13, 35]. While the SECOMBIT trial showed that a brief course of BRAF/MEK inhibition may enhance the OS outcomes for a subset of patients, given that this effect was largely limited to the population of patients with an elevated baseline LDH [31] suggests that this result is related to effects on multiple components of the TIME rather than the observed changes in immune cells observed in our patients.

## Author Contributions

**Suthee Rapisuwon:** conceptualization (equal), data curation (equal), formal analysis (equal), investigation (equal), writing – original draft (lead), writing – review and editing (equal). **Craig L. Slingluff Jr.:** conceptualization (equal), data curation (equal), formal analysis (equal), funding acquisition (equal), investigation (equal), writing – review and editing (equal). **Jennifer A. Wargo:** investigation (equal), writing – review and editing (equal). **Ryan J. Sullivan:** investigation (equal), writing – review and editing (equal). **Benjamin Izar:** investigation (equal), writing – review and editing (equal). **Jia‐Ren Lin:** formal analysis (equal), writing – review and editing (equal). **Ileana S. Mauldin:** investigation (equal), writing – review and editing (equal). **Walter C. Olson:** investigation (equal), writing – review and editing (equal). **Christine A. Tran:** investigation (equal), writing – review and editing (equal). **Gabrielle H. Schwartzman:** investigation (equal), writing – review and editing (equal). **Geoffrey T. Gibney:** investigation (equal), writing – review and editing (equal). **Waddah Al‐Refaie:** investigation (equal), writing – review and editing (equal). **Michael B. Atkins:** conceptualization (lead), funding acquisition (equal), investigation (equal), writing – review and editing (lead).

## Funding

Funding for the clinical trial was provided by Genentech (PI MBA), and funding for the correlative studies was provided by a Team Science grant from the Melanoma Research Alliance (MBA, CS). US Public Health Services Grants P30 CA044579 (Molecular and Immunologic Translational Sciences Core, Biorepository and Tissue Research Facility, Office of Clinical Research) (GHS), P30 CA051008 (Deputy Director, Histopathology and Tumor Share Resource, Flow Cytometry Shared Resource, Genomics and Epigenomic Shared Resource, and Biostatistics Shared Resource) (MBA); T32 CA163177 (CAT); Rebecca Clary Harris, MD Memorial Fellowship (GHS).

## Ethics Statement

The clinical trial and embedded biomarker studies were approved by the FDA (IND#124857), the Georgetown‐MedStar Cancer Institutional Review Board, and subsequently the Institutional Review Boards at the other participating institutions (University of Virginia, Massachusetts General Hospital, and MD Anderson Cancer Center). Written informed consent was obtained from each patient.

## Conflicts of Interest

Suthee Rapisuwon has the following disclosures (none related to this project): Research support to Georgetown University from Bristol Myers Squibb (funding, drug). He also has/had an advisory role for Castle Bioscience and Replimune. He holds stocks in Cardiff Oncology and Geron Corporation. Craig L. Slingluff has the following disclosures (none related to this project): Research support to the University of Virginia from Celldex (funding, drug), Merck (funding, drug), 3 M (drug), Theraclion (device staff support); Funding to the University of Virginia from Polynoma for PI role on the MAVIS Clinical Trial; Funding to the University of Virginia for roles on Scientific Advisory Boards for Immatics and CureVac. Also Dr. Slingluff receives licensing fee payments through the UVA Licensing and Ventures Group for patents for peptides used in cancer vaccines. Jennifer A. Wargo is co‐inventor on a patent submitted by The University of Texas MD Anderson Cancer Center to the US Patent and Trademark Office on modulating the microbiome to enhance responses to immune checkpoint blockade (Patent # PCT/US1/53717), and on another patent Targeting B Cells To Enhance Response To Immune Checkpoint Blockade UTSC.P1412US.P1—MDA19‐023. She also is on advisory boards for Gustave Roussy Cancer Center and for the Ohio State University Comprehensive Cancer Center. Ryan J. Sullivan has received consulting fees from Marengo, Merck, Novartis, Pfizer, and Replimune; and has received research support from Merck. Benjamin Izar has received consulting fees/honoraria from Volastra Therapeutics Inc., Merck, AstraZeneca, Novartis, Eisai, and Janssen Pharmaceuticals and has received research funding to Columbia University from Alkermes, Arcus Biosciences, Checkmate Pharmaceuticals, Compugen, Immunocore, Merck, Regeneron, and Synthekine. He is a founder of Basima Therapeutics Inc. Jia‐Ren Lin—None. Ileana S. Mauldin—None. Walter C. Olson—None. Christine A. Tran—None. Gabrielle H. Schwartzman—None. Geoffrey T. Gibney has an advisory role for Bristol Myer‐Squibb, Huyabio, Immunocore, Iovance, Lyell, Merck, Novartis, Pfizer, Regeneron, and Replimune; research support (institutional) from Exelixis, and is on the speaker's bureau for Immunocore. Waddah Al‐Refaie—None. Michael B. Atkins has/had an advisory role for 23ANDME, AbbVie, Agenus, Asher Bio, AstraZeneca, Atreca, Aveo, Beigene, Boehringer‐Ingelheim, Bristol Myers Squibb, Eisai, Exelixis, GSK, IO Biotech, Innovent, JAZZ Pharmaceuticals, Merck, Novartis, OncoRena, Pfizer, Pliant Therapeutics, Pyxis Oncology, Roche, SAB Bio, Sanofi, SeaGen, Simcha, Replimune, Syncona, Takeda, Werewolf Therapeuctics. He holds stock in Pyxis Oncology and Werewolf Therapeutics and is on the Board of Directors for Werewolf Therapeutics. None are related to this project.

## Supporting information


**Data S1:** cam471526‐sup‐0001‐TableS1‐S6.docx.

**Data S2:** cam471526‐sup‐0002‐TableS2‐S25.xlsx.

**Data S3:** cam471526‐sup‐0003‐FigureS3‐S25.docx.

## Data Availability

The PIs are committed to the concept that data sharing is essential for expedited translation of research results. Members of our groups will adhere to the policy that the timely release and sharing of data obtained from the research will be made available at the time of publication. Members of our group will also share results from our studies at national meetings and with other investigators based on their availability. Data will be kept free of identifiers that would permit linkages to individual patients and variables that could lead to disclosure of their identity.
