# Supplementary material for: Effects of Vemurafenib ± Cobimetinib on Intratumoral and Host Immunity in Patients With BRAFV600 Mutant Melanoma: Implications for Combination With Immunotherapy
Source: Cancer Med. 2026 Jan 9;15(1):e71526. doi: 10.1002/cam4.71526 (PMC12789045; doi:10.1002/cam4.71526)
Supplement: Supplementary file 1 — Data S1: cam471526‐sup‐0001‐TableS1‐S6.docx. [file CAM4-15-e71526-s003.docx]

**Supplemental Table 1: Antibodies Used in Flow Cytometry Analyses**

| Ab Specificity | Clone | Species | Fluorescent tag | Source | Catalog # | Notes |
| --- | --- | --- | --- | --- | --- | --- |
| CD4 | RPA-T4 | mIgG1 | PE | Biolegend | 555347 | 1:50 |
| CD45RO | UCHL1 | mIgG2a | FITC | Biolegend | 304204 | 1:50 |
| Ki67 | Ki67 | mIgG1 | PE-Cy7 | Biolegend | 350526 | 1:100 |
| CD3 | UCHT1 | mIgG1 | v450 | BD Biosciences | 560365 | 1:50 |
| CD3 | SK7 | mIgG1 | APC | BD Biosciences | 570820 | 1:50 |
| CD3 | SK7 | mIgG1 | APC-Cy7 | BD Biosciences | 560275 | 1:50 |
| CD4 | RPA-T4 | mIgG1 | Fitc | BD Biosciences | 555346 | 1:50 |
| CD8 | RPA-T8 | mIgG1 | PE-Cy7 | BD Biosciences | 557750 | 1:100 |
| CD8 | SK1 | mIgG1 | APC-H7 | BD Biosciences | 561423 | 1:100 |
| CD27 | M-T271 | mIgG1 | PE | BD Biosciences | 555441 | 1:50 |
| CD28 | Cd28.2 | mIgG1 | APC | BD Biosciences | 559770 | 1:50 |
| CD45RA | HI 100 | mIgG2b | V450 | BD Biosciences | 560362 | 1:50 |
| HLA-DR | G46.6 | mIgG2z | APC-H7 | eBioscience | 561358 | 1:100 |
| CD127 | eBioRDR5 | mIgG1 | PerCP-Cy5.5 | eBioscience | 45-1278-42 | -- |
| CD25 | BC96 | mIgG1 | PE | eBioscience | 12-0259-41 | 1:40 |
| Rat IgG2a | eBR2a | Rat IgG2a | APC | eBioscience | 17-4321-81 | 1:50 |
| FoxP3 | PUH101 | Rat IgG2a | APC | eBioscience | 17-4776-42 | 1:50 |
| EOMES | WD1928 | mIgG1 | PE | eBioscience | 12-4877-42 | 1:50 |
| PD-1 | Ebio1185 | mIgG1 | PerCP-ef710 | eBioscience | 46-2799-41 | 1:50 |
| ICOS-1/CD278 | ISA-3 | mIgG1 | APC-ef780 | Invitrogen | 47-9948-42 | 1:50 |
| Tbet | Ebio4B10 | mIgG1 | PE-Cy7 | Invitrogen | 25-5825-82 | 1:50 |
| CD19 | HIB19 | mIgG1 | Fitc | BD Biosciences | 555412 | 1:100 |
| CD14 | M5E2 | mIgG2a | PerCP-Cy5.5 | BD Biosciences | 561115 | 1:100 |
| CD56 | Tuly56 | mIgG1 | APC | Invitrogen | 17-0566-42 | 1:50 |
| CCR7 | 150503 | mIgG2a | PerCP-Cy5.5 | BD Biosciences | 561144 | 1:50 |
| TIM-3 | F28-2E2 | mIgG1 | BV421 | Invitrogen | 345008 | 1:50 |

**Supplemental Table 2: Grade 3+ Treatment-Emergent Adverse Events**

| **Toxicity** | **Grade** | **Number (%)** |
| --- | --- | --- |
| ALT | 3 | 2 (40%) |
| AST | 3 | 1 (20%) |
| Dehydration | 3 | 1 (20%) |
| Headache | 3 | 2 (40%) |
| Hyperuricemia | 3 | 1 (20%) |
| Lymphopenia | 3 | 3 (60%) |
| Nausea | 3 | 2 (40%) |
| Pain in Extremity | 3 | 1 (20%) |
| Photosensitivity | 3 | 1 (20%) |
| Rash – maculo-papular | 3 | 1 (20%) |
| Sub-retinal edema | 3 | 1 (20%) |
| Vomiting | 3 | 2 (40%) |

**Supplemental Table 2**: The list of grade 3 treatment-emergent adverse events (TRAEs) were observed. All except one headache and one each nausea/vomiting/dehydration were deemed to be treatment related. No grade 4 TRAEs or treatment-related deaths were observed on-study.

**Supplemental Table 3: Genes with significantly altered expression during BRAFi/MEKi therapy**

[see separate table in Excel]: too large for here.

**Supplemental Table 4. BioPlanet Gene Pathways significantly altered with BRAFi/MEKi therapy**

| **Term Name** |  | **D8 vs D1** | | | **D15 vs D1** | | | **D29 vs D1** | | | |
| --- | --- | --- | --- | --- | --- | --- | --- | --- | --- | --- | --- |
|  | **# Genes in Term** | **p-adj** | **# genes** | | **p-adj** | **# genes** | | **p-adj** | **# genes** | | |
|  |  |  | **Up** | **Down** |  | **Up** | **Down** |  | **Up** | **Down** |  |
| Adaptive immune system | 606 | 0.0001 | 57 | 1 | 0.0002 | 68 | 1 | <0.0001 | 62 | 1 |  |
| Antigen processing and presentation | 81 | 0.0022 | 22 | 0 | 0.0023 | 25 | 0 | 0.0003 | 24 | 0 |  |
| Viral myocarditis | 71 | 0.0057 | 19 | 0 | 0.0033 | 22 | 0 | 0.0004 | 21 | 0 |  |
| Cell adhesion molecules (CAMs) | 133 | 0.0084 | 29 | 1 | 0.0044 | 35 | 1 | 0.0004 | 33 | 1 |  |
| Immune system | 998 | 0.0133 | 98 | 4 | 0.0002 | 129 | 6 | 0.0010 | 107 | 6 |  |
| Antigen processing: cross presentation | 79 | 0.0162 | 14 | 0 | 0.0144 | 16 | 0 | 0.0033 | 15 | 0 |  |
| Allograft rejection | 37 | 0.0180 | 18 | 0 | 0.0151 | 21 | 0 | 0.0008 | 21 | 0 |  |
| Graft-versus-host disease | 41 | 0.0180 | 18 | 0 | 0.0151 | 21 | 0 | 0.0003 | 22 | 0 |  |
| Type 1 diabetes mellitus | 43 | 0.0207 | 17 | 0 | 0.0151 | 20 | 0 | 0.0003 | 21 | 0 |  |
| T cell receptor regulation of apoptosis | 603 | 0.0213 | 50 | 4 | 0.0261 | 63 | 3 | 0.0017 | 56 | 5 |  |
| Endosomal/vacuolar pathway | 9 | 0.0220 | 6 | 0 | -- | -- | -- | 0.0223 | 6 | 0 |  |
| PD-1 signaling | 31 | 0.0249 | 12 | 0 | 0.0177 | 14 | 0 | 0.0017 | 14 | 0 |  |
| MHC class II antigen presentation | 103 | 0.0294 | 10 | 0 | 0.0144 | 12 | 0 | 0.0014 | 12 | 0 |  |
| Autoimmune thyroid disease | 52 | 0.0294 | 18 | 0 | -- | -- | -- | 0.0063 | 20 | 0 |  |
| Interleukin-12/STAT4 pathway | 45 | 0.0439 | 18 | 0 | -- | -- | -- | -- | -- | -- |  |
| Antigen presentation: folding, assembly, and peptide loading of class I MHC proteins | 255 | 0.0445 | 14 | 0 | 0.0236 | 17 | 0 | 0.0063 | 16 | 0 |  |
| T cell activation co-stimulatory signal | 67 | 0.0491 | 13 | 0 | -- | -- | -- | 0.0438 | 10 | 0 |  |
| Generation of second messenger molecules | 36 | 0.0491 | 10 | 0 | 0.0261 | 12 | 0 | 0.0032 | 12 | 0 |  |
| Costimulation by the CD28 family | 72 | 0.0491 | 15 | 0 | 0.0405 | 18 | 0 | 0.0030 | 18 | 0 |  |
| Phagosome | 154 | -- | -- | -- | 0.0002 | 34 | 0 | 0.0018 | 28 | 0 |  |
| Systemic lupus erythematosus | 139 | -- | -- | -- | -- | -- | -- | 0.0224 | 21 | 0 |  |

**Supplemental Table 5. Nanostring- Significant adjusted gene expression changes in cell subsets**

|  |  | **D8 vs D1** | | | **D15 vs D1** | | | **D29 vs D1** | | |
| --- | --- | --- | --- | --- | --- | --- | --- | --- | --- | --- |
| Term Name | # Genes  in  Term | FDR-  adjusted  p-Value | Genes  up | Genes  down | FDR-  adjusted  p-Value | Genes  up | Genes down | FDR-  adjusted  p-Value | Genes up | Genes down |
| CD8+ T cells | 577 | 0.00015 | 33 | 0 | 0.00982 | 34 | 0 | 0.00336 | 31 | 0 |
| CD14+ Monocytes | 374 | 0.00018 | 29 | 1 | 5.30E-06 | 37 | 1 | 2.00E-06 | 34 | 1 |
| Whole Blood | 507 | 0.00151 | 34 | 2 | 0.01585 | 40 | 0 | 0.01334 | 35 | 0 |
| CD33+ Myeloid | 660 | 0.00559 | 32 | 3 | 0.00079 | 43 | 2 | 0.00063 | 38 | 2 |
| CD4+ T cells | 515 | 0.00633 | 25 | 0 | -- | -- | -- | -- | -- | -- |
| CD56+ NK Cells | 728 | 0.00633 | 34 | 0 | -- | -- | -- | -- | -- | -- |
| BDCA4+ DCs | 482 | 0.01572 | 23 | 0 | 0.01585 | 28 | 0 | 0.01334 | 25 | 0 |

**Supplemental Table 6 – Productive Frequencies of Significantly Increased T Cells Per Clonotype by Treatment Day**

| **Patient** | **TCR Sequence of Vβ CDR3 region of increasing clonotypes** | **Day 1** | **Day 8** | **Day 15** | **Day 29** | **Day 35** |
| --- | --- | --- | --- | --- | --- | --- |
| 2 | CTGGAGTCCGCCAGCACCAACCAGACATCTATGTACCTCTGTGCCAGCAGCCGAGGACAGGGCCGTGAAAAACTGTTTTTTGGCAGT | 0.00241 | 0.00283 | 0.05434* | 0.00974 | 0.0100 |
| 3 | CTGAGCTCTCTGGAGCTGGGGGACTCAGCTTTGTATTTCTGTGCCAGCAGCGTAGCACCGGGACAGGGAATATACACCTTCGGTTCG | 0.00232 | 0.00604 | 0.04467* | 0.02933* | - |
|  | GCTACCAGCTCCCAGACATCTGTGTACTTCTGTGCCATCAGTGAGTTAGGGCTGGGGCCCTCTTCCTACGAGCAGTACTTCGGGCCG | 0.01392 | 0.00604 | 0.01873 | 0.03888* | - |
|  | CGCACAGAGCGGGGGGACTCAGCCGTGTATCTCTGTGCCAGCAGCTTGAGTACAGGGGTTATTCCAGATACGCAGTATTTTGGCCCA | 0 | 0.00302 | 0.00144 | 0.01160* | - |
| 5 | CTTCACCTACACGCCCTGCAGCCAGAAGACTCAGCCCTGTATCTCTGCGCCAGCAGCCAAGATGGGAACGAGCAGTACTTCGGGCCG | 0 | 0.11295* | 0.02875* | 0.00316 | - |
|  | ACAGTGACCAGTGCCCATCCTGAAGACAGCAGCTTCTACATCTGCAGTCCTGGCATCGGGAGGTCAGATACGCAGTATTTTGGCCCA | 0 | 0.08558* | 0 | 0.00158 | - |
|  | AAGATCCAGCCCTCAGAACCCAGGGACTCAGCTGTGTATTTTTGTGCTAGTGGTTTGCCCACCGGAGTCGAGCAGTACTTCGGGCCG | 0 | 0.06584* | 0.04152* | 0.00316 | - |
|  | CTGAGCTCTCTGGAGCTGGGGGACTCAGCTTTGTATTTCTGTGCCAGCAGCGTAGAAGGTAACACCGGGGAGCTGTTTTTTGGAGAA | 0.00219 | 0.07232* | 0.06739* | 0.13880* | - |
|  | GAGTCGCCCAGCCCCAACCAGACCTCTCTGTACTTCTGTGCCAGCACGATAACGGGGGGCTTTAATTCACCCCTCCACTTTGGGAAC | 0 | 0.04898* | 0.00256 | 0.00158 | - |
|  | TCAGAACCGGGAGACACGGCACTGTATCTCTGCGCCAGCAGTCAGTCGGGACAGGGAAGTTCTGGAAACACCATATATTTTGGAGAG | 0.00132 | 0.04610* | 0.03929* | 0.10568* | - |
|  | ATGAGCTCCTTGGAGCTGGGGGACTCAGCCCTGTACTTCTGTGCCAGCAGCTTAGGCCTCTCGGGGGGACCTGCGTCTTTTGGCCCA | 0.00263 | 0.04769* | 0.01821* | 0.00788 | - |
|  | CTGGGGTTGGAGTCGGCTGCTCCCTCCCAAACATCTGTGTACTTCTGTGCCAGCAGTTTAGGGGCCTACGAGCAGTACTTCGGGCCG | 0 | 0.03515* | 0.00607* | 0.00473 | - |
|  | ACCAGTGCCCATCCTGAAGACAGCAGCTTCTACATCTGCAGTGCTAGTGATCAGGGAATTGGGGCCAACGTCCTGACTTTCGGGGCC | 0 | 0.01354* | 0.03417* | 0.01893* | - |
|  | ATCCAGCCCTCAGAACCCAGGGACTCAGCTGTGTACTTCTGTGCCAGCAGGACCGGGACACCCTTTACCAAGCAGTACTTCGGGCCG | 0 | 0.00908* | 0.00192 | 0 | - |
|  | AATGTGAGCACCTTGGAGCTGGGGGACTCGGCCCTTTATCTTTGCGCCAGCACGGGGGTCAGCTCCTACGAGCAGTACTTCGGGCCG | 0 | 0.00490* | 0.00830* | 0.00158 | - |
|  | AGCACCTTGGAGCTGGGGGACTCGGCCCTTTATCTTTGCGCCAGCAGCTTGGGGGACTACAGTAACACTGAAGCTTTCTTTGGACAA | 0 | 0.00375* | 0.00128 | 0 | - |
|  | GTGAGCACCTTGGAGCTGGGGGACTCGGCCCTTTATCTTTGCGCCAGCAGCTTGGCCACAGGGAACACTGAAGCTTTCTTTGGACAA | 0 | 0.00317* | 0 | 0.02681* | - |
|  | GAGTCTGCCAGGCCCTCACATACCTCTCAGTACCTCTGTGCCAGCAGTGAATATAACGGTGAACACAGTGAAGCTTTCTTTGGACAA | 0 | 0.00317* | 0 | 0.00158 | - |
|  | GGGTTGGAGTCGGCTGCTCCCTCCCAAACATCTGTGTACTTCTGTGCCAGCAGTTTTGGCAATGAGGCTGAAGCTTTCTTTGGACAA | 0.00088 | 0.00475* | 0.00096 | 0.01420* | - |
|  | TTGGAGTCGGCTGCTCCCTCCCAAACATCTGTGTACTTCTGTGCCAGCAGTTACGGGACTAGCGGGGATTGGACGTACTTCGGGCCG | 0.00175 | 0.00576* | 0.00575* | 0.00631 | - |
|  | ACAGTGACCAGTGCCCATCCTGAAGACAGCAGCTTCTACATCTGCAGTGCCCGACAGGGGGCAGCAGATACGCAGTATTTTGGCCCA | 0 | 0.00231* | 0.00128 | 0 | - |
|  | ACTGTGACATCGGCCCAAAAGAACCCGACAGCTTTCTATCTCTGTGCCAGTAGTATTGAAGGTATCACTGAAGCTTTCTTTGGACAA | 0 | 0.00043 | 0.16959* | 0 | - |
|  | GAGTCCGCCAGCACCAACCAGACATCTATGTACCTCTGTGCCAGCAGTGAGGCAAGGGCAGGGATAGATACGCAGTATTTTGGCCCA | 0 | 0 | 0.02491* | 0 | - |
|  | AAGCTCCTCCTCAGTGACTCTGGCTTCTATCTCTGTGCCTGGAGTTCCGGGACAGGGGGCATAGGAGATACGCAGTATTTTGGCCCA | 0 | 0.00159 | 0.01629* | 0.00158 | - |
|  | ACTGTGAGCAACATGAGCCCTGAAGACAGCAGCATATATCTCTGCAGCGTTGACCCAGGGGGCTTCTATGGCTACACCTTCGGTTCG | 0 | 0.00029 | 0.00894* | 0 | - |
|  | ATCCAGCCCTCAGAACCCAGGGACTCAGCTGTGTACTTCTGTGCCAGCAGCCTGGGGGGACCGGGCCAGCCCCAGCATTTTGGTGAT | 0 | 0.00029 | 0.00767* | 0 | - |
|  | ATCCAGCGCACAGAGCAGCGGGACTCGGCCATGTATCGCTGTGCCAGCAGCCCGTGGGACCGGGCGTACGAGCAGTACTTCGGGCCG | 0 | 0.00029 | 0.00639* | 0 | - |
|  | AACCTGAGCTCTCTGGAGCTGGGGGACTCAGCTTTGTATTTCTGTGCCAGCAGCGCGGGACAGATCAACATTCAGTACTTCGGCGCC | 0.00175 | 0.00475 | 0.00703* | 0.00316 | - |
|  | CTGGGGTTGGAGTCGGCTGCTCCCTCCCAAACATCTGTGTACTTCTGTGCCAGCAGTTACGGGGCGTACGAGCAGTACTTCGGGCCG | 0 | 0.00130 | 0.00319* | 0 | - |
|  | CGCACACAGCAGGAGGACTCGGCCGTGTATCTCTGTGCCAGCAGCCAGGGAGCGGGGCCCGGGACAGATACGCAGTATTTTGGCCCA | 0.00132 | 0.00303 | 0.00607* | 0.00316 | - |
|  | ATCCAGCAGGTAGTGCGAGGAGATTCGGCAGCTTATTTCTGTGCCAGCTCACCAGACAGGGTGGGCCAGCCCCAGCATTTTGGTGAT | 0.00132 | 0.00245 | 0.00607* | 0.00158 | - |

* Significantly increased (p < 0.05) compared to Day 1 pre-treatment sample.

- No evaluable tumor samples.
