# Supplementary material for: Effects of Vemurafenib ± Cobimetinib on Intratumoral and Host Immunity in Patients With BRAFV600 Mutant Melanoma: Implications for Combination With Immunotherapy
Source: Cancer Med. 2026 Jan 9;15(1):e71526. doi: 10.1002/cam4.71526 (PMC12789045; doi:10.1002/cam4.71526)
Supplement: Supplementary file 3 — Data S3: cam471526‐sup‐0003‐FigureS3‐S25.docx. [file CAM4-15-e71526-s002.docx]

**Supplemental Figures**


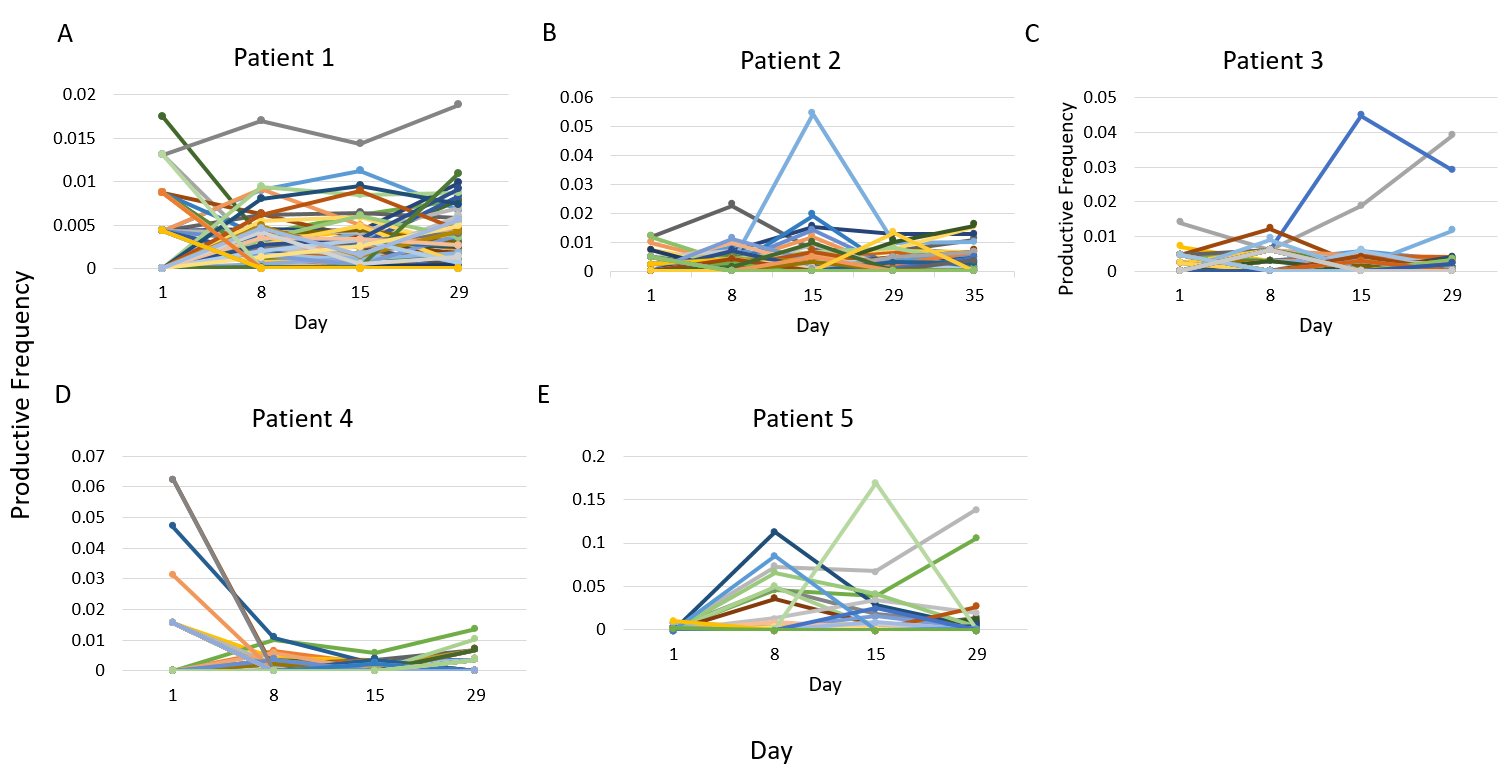


**Supplemental Figure 1. Top 25 Track Rearrangements by Productive Frequency –** Trends of the top 25 track rearrangements by productive frequency for each patient by tumor biopsy day. For each patient, the productive frequencies of all graphed tracked rearrangements do not add up to 1 as only the top 25 track rearrangements by productive frequency are graphed. For an individual patient, each T cell clonotype is represented by a unique color.


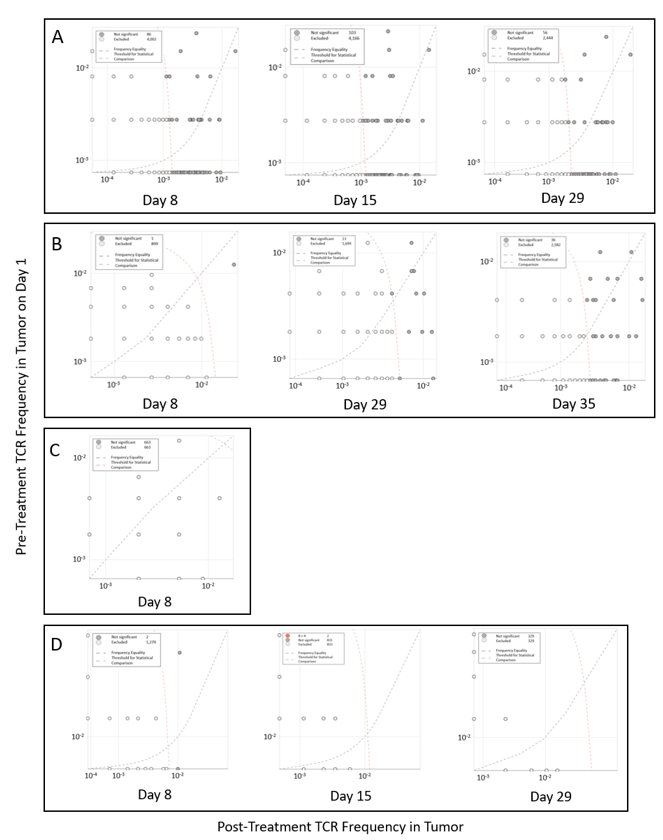


**Supplemental Figure 2. Comparison of TCR Clonotypes in Tumor Pre- vs. Post-Treatment. For those samples without significant clonal expansion of TCR Vβ, frequencies of each TCR Vβ are shown for each time point compared to baseline. F**requency equality pre-treatment vs. post-treatment is represented by the gray dotted oblique line. The threshold for statistical comparison is represented by the red dotted curved line. Sequences represented by light gray were below this threshold and subsequently excluded from analysis. Sequences represented by orange appear in higher frequency pre-treatment than post-treatment. Sequences that appear on the x-axis are present post-treatment, but absent pre-treatment. Both of these scenarios suggest that TILs appear in higher frequency post-treatment vs. pre-treatment. Sequences represented by dark gray are not statistically significant (p > 0.05). A) Patient 1 – Day 1 vs. Days 8, 15, and 29; B) Patient 2 – Day 1 vs. Days 8, 29, and 35; C) Patient 3 – Day 1 vs. Day 8; D) Patient 5 – Day 1 vs. Days 8, 15, and 29.


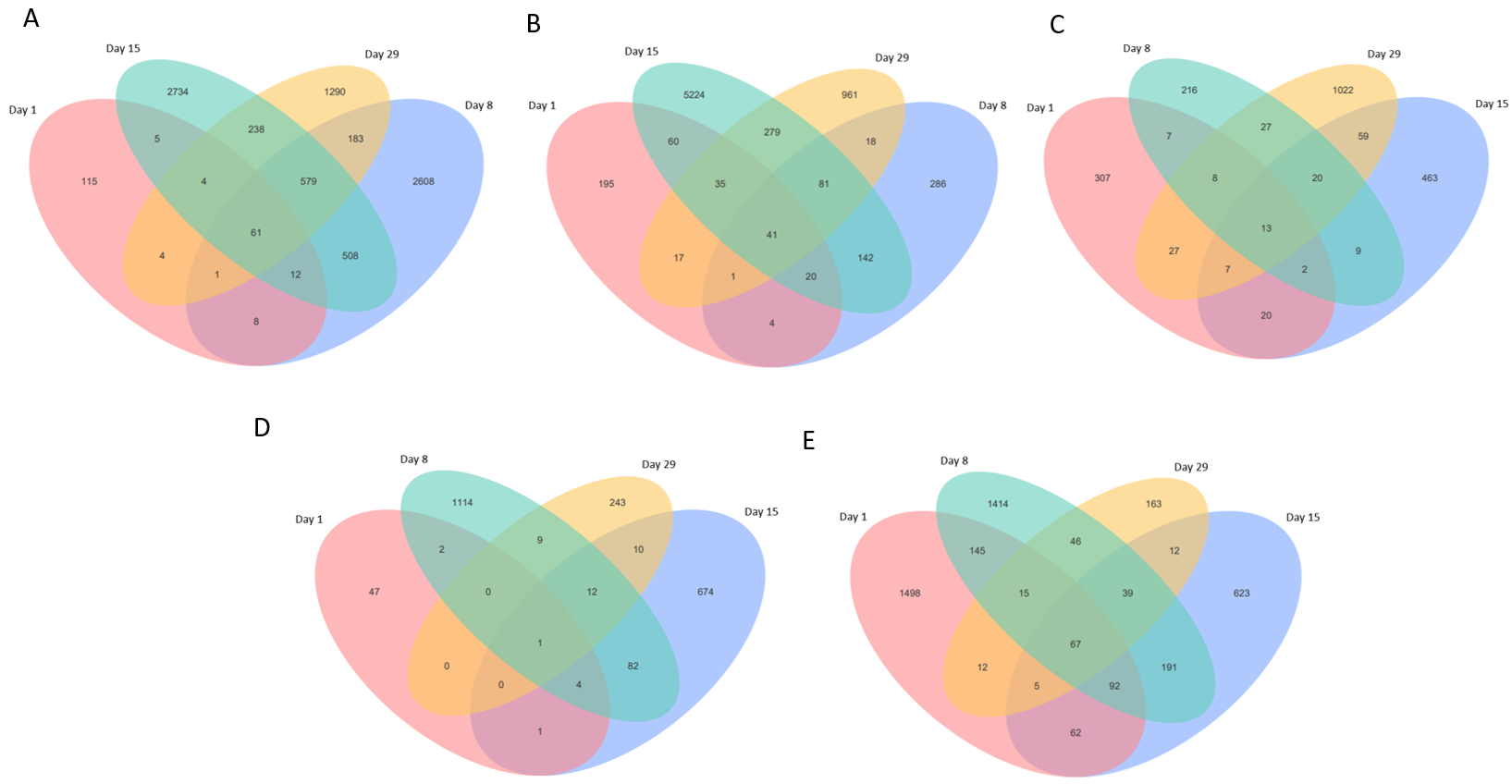


**Supplemental Figure 3.** **Number of Overlapping TCR Clonotypes by Treatment Day** – The number of overlapping TCR clonotypes by treatment day for each patient was plotted on a Venn diagram. A-E) Patients 1-5, respectively.

**A**


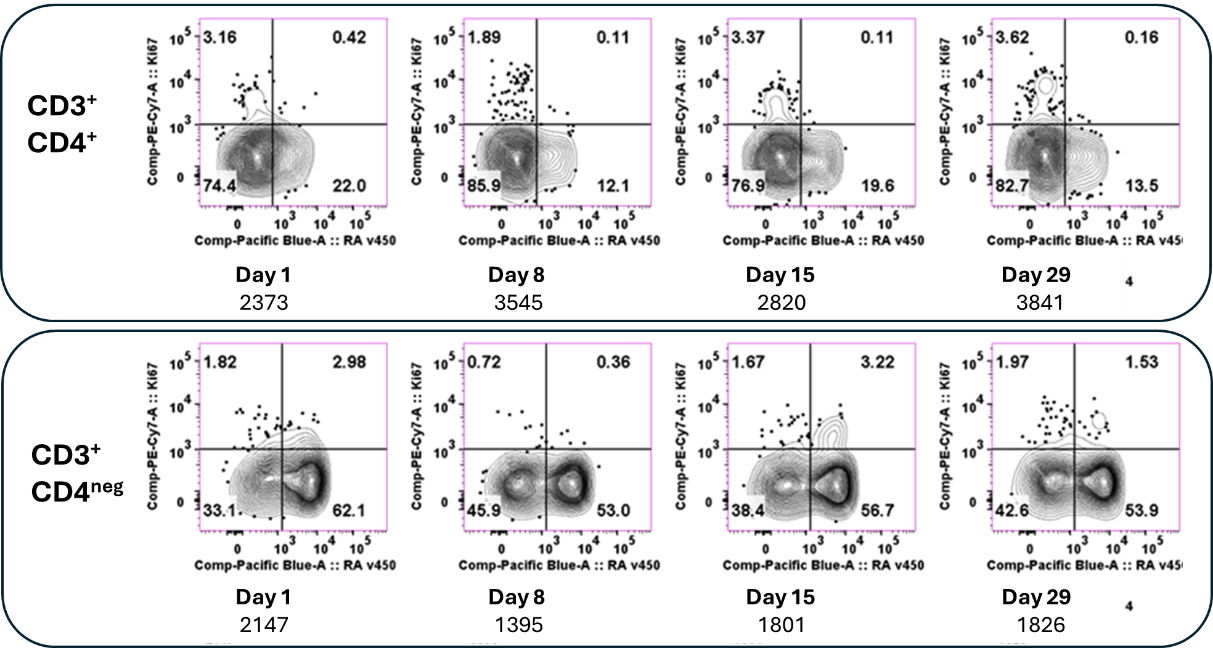


**B**


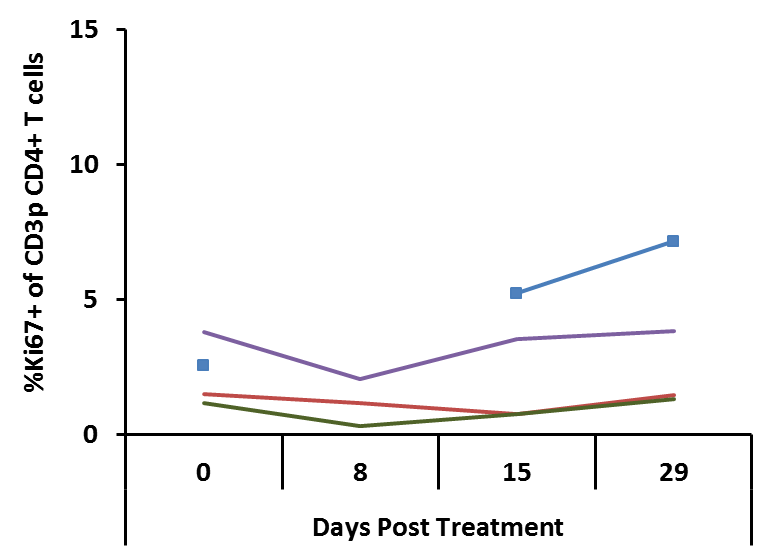

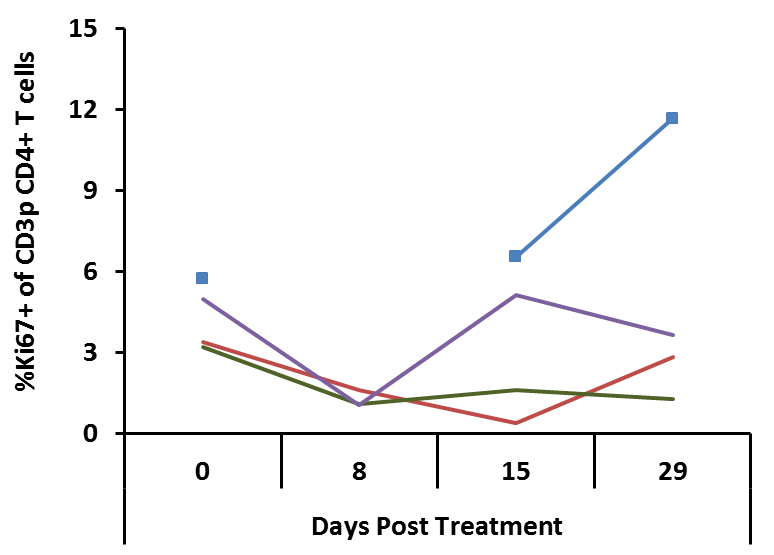

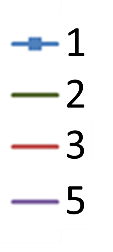


**Supplemental Figure 4. Evaluation of circulating CD4 and CD8 T cells for changes in proliferation with BRAFi/MEKi.**  Fluorescent activated cell sorting of peripheral blood T cells at baseline, and days 8, 15, and 29. (A) Representative data (subject #5) for markers of antigen experience (CD45RA as not antigen experienced) and proliferation (Ki67) for cells gated for CD3, CD4, and CD8 (CD3+CD4-). (B) Summary data for proliferation of CD4 and CD8 T cells for 4 evaluable patients. The d8 sample for subject 1 was insufficient for analysis.
